# Impact of Reed Canary Grass Cultivation and Mineral Fertilisation on the Microbial Abundance and Genetic Potential for Methane Production in Residual Peat of an Abandoned Peat Extraction Area

**DOI:** 10.1371/journal.pone.0163864

**Published:** 2016-09-29

**Authors:** Mikk Espenberg, Marika Truu, Jaak Truu, Martin Maddison, Hiie Nõlvak, Järvi Järveoja, Ülo Mander

**Affiliations:** Department of Geography, Institute of Ecology and Earth Sciences, University of Tartu, Tartu, Estonia; RMIT University, AUSTRALIA

## Abstract

This study examined physiochemical conditions and prokaryotic community structure (the bacterial and archaeal 16S rRNA genes and *mcrA* gene abundances and proportions), and evaluated the effect of reed canary grass cultivation and mineral fertilisation on these factors, in the 60 cm thick residual peat layer of experimental plots located on an abandoned peat extraction area. The archaeal proportion was 0.67–39.56% in the prokaryotic community and the methanogens proportion was 0.01–1.77% in the archaeal community. When bacterial abundance was higher in the top 20 cm of peat, the archaea were more abundant in the 20–60 cm layer and methanogens in the 40–60 cm layer of the residual peat. The bacterial abundance was significantly increased, but archaeal abundance was not affected by cultivation. The fertiliser application had a slight effect on peat properties and on archaeal and methanogen abundances in the deeper layer of cultivated peat. The CH_4_ emission was positively related to *mcrA* abundance in the 20–60 cm of the bare peat, while in case of reed canary grass cultivation these two parameters were not correlated. Reed canary grass cultivation mitigated CH_4_ emission, although methanogen abundance remained approximately the same or even increased in different layers of residual peat under cultivated sites over time. This study supports the outlook of using abandoned peat extraction areas to produce reed canary grass for energy purposes as an advisable land-use practice from the perspective of atmospheric impact in peatland-rich Northern Europe.

## Introduction

Peatlands cover about 3% of the world’s land area [1]. These ecosystems play a vital role in carbon accumulation and long-term sequestration and store ⅓ of the world’s soil carbon, hence they have a great impact on global climate change [1,2]. Peatlands can be classified on the basis of anthropogenic impact as undisturbed, drained and mined [3]. Modern peat mining is conducted using large-scale milling and vacuum removal of recently dried peat along with clearance of the surface vegetation [4].

In peatland-rich regions (North America, Northern Europe), the extracted peat is widely used as an energy source or as a growing medium and a soil conditioner in horticulture [5–7]. In addition, there are various other areas (including medicine, textile and construction industries) where this material is used [1]. The high demand for this material leads to the intensive extraction of peat and inevitably creates environmental problems concerning abandoned peatlands.

Once abandoned, cutover peatlands expose well-decomposed peat at the surface with extremely harsh environmental conditions such as altered hydrology, wind erosion, frost heaving and variable physicochemical properties [8], whereby the reduced microbial activity limits nutrient replenishment [9]. Consequently, plant colonisation and growth are also hindered in this kind of substrate [8]. Thus, several environmental issues emerge including the negative impact on the surrounding hydrologic system, risk of fires, biological and landscape diversity loss and continuous greenhouse gas (CO_2_, CH_4_, N_2_O) emissions into the atmosphere [6,10]. Therefore, it is necessary to restore abandoned peatland as closely as possible back to their natural state [10] or to use them for other purposes, such as bioenergy production [11,12], agricultural land and berry cultivation [13] and afforestation of land [14].

Since microbially mediated greenhouse gas production and also reduction processes take place in peat [5], the implementation of peatlands management approaches that also take into account the specific aspects of methane (CH_4_) production and oxidation are crucial. In the assessment of global warming potential, understanding the dynamics of CH_4_ emissions is a central issue, while CH_4_ is the second most prevalent anthropogenic greenhouse gas. However, there is still much uncertainty surrounding the mechanism (a complex set of microbial, plant and soil physicochemical factors) regulating fluxes of CH_4_ from different soils into the atmosphere [15]. CH_4_ is generated through anaerobic degradation of organic matter by methanogenic archaea. Although these organisms are of great phylogenetic and also ecological diversity [16], all methanogens that have been characterised so far possess *mcrA* gene, which encodes the alpha-subunit of the methyl coenzyme M reductase—the enzyme that catalyses the last step in the CH_4_ synthesis converting the fermentation end products (e.g. H_2_/CO_2_ and acetate) to CH_4_ [15,17].

Using abandoned peat extraction areas to produce biomass, such as reed canary grass, for energy purposes has been suggested as an option to mitigate atmospheric impact in peatland-rich Northern Europe [12,18,19]. However, our knowledge of belowground processes in different types of peatlands has remained fragmentary [10,20,21] and little is known about microbial communities and their relationships with plant establishment, carbon accumulation and greenhouse gas emissions in residual peat [10].

The aim of this study was to assess the effect of reed canary grass cultivation and fertilisation on prokaryotic community abundance (bacterial and archaeal and methanogenic archaeal abundance and proportion) in residual peat on an abandoned peat extraction area, and link these changes to methane emission from the peat.

## Materials and Methods

### Site description and peat sampling

The experiment was conducted in the period from June 2012 to September 2014 in the Lavassaare cutover peatland complex (58°34'20'' N, 24°23'15'' E) that is the largest peat extraction area (19,746 ha) in Estonia. The mean annual air temperature and precipitation were 5.6°C and 985 mm, and 7.0°C and 637 mm in 2012 and 2014, respectively. The residual peat column was variable (0.3–1.2 m) across the area and consisted primarily of well mineralised *Phragmites-Carex* peat (H7 class according to the von Post decomposition scale). The area was divided into 20 m wide strips by the drainage ditches [12,19] and some of the abandoned peat extraction strips were sowed with reed canary grass (*Phalaris arundinacea* L. Estonian-bred variety “Pedja”) in the spring of 2007.

Three cultivated and uncultivated peat strips were chosen for the experiment. On each strip, one fertilised and one control plot (2.5 x 10 m) with 0.5–0.7 m peat layer were set up. All the plots were located at 4 m distance from ditches. The distance between control and fertilised plot was at least 4 m on a peat strip. Based on chemical analysis of the study site peat, 72 kg N, 18 kg P and 36 kg K of mineral fertiliser was applied per hectare once per year (in June) on fertilised plots.

The peat samples were collected before the first fertilisation (June 2012), at the end of the first vegetation period (September 2012) and after the third vegetation period (September 2014). Each plot was divided into two equal subareas and three separate 60 cm cores for each subarea were taken using a peat corer (Ø 5cm). The cores were cut into three 20 cm vertical segments (0–20, 20–40 and 40–60 cm) and three segments from the same layer of one subarea were pooled to make a composite sample. In total, 216 composite peat samples were collected during the three sampling campaigns. In the laboratory, the samples were homogenised and divided into subsamples for chemical and molecular analyses. Sub-samples for molecular analyses were stored at −20°C and for chemical analyses at +4°C. The chemical analyses were performed in the Estonian Environmental Research Centre. Peat pHH_2_O (pH) and dissolved organic carbon (DOC), Kjedahl nitrogen (TN), ammonium nitrogen (NH_4_-N), nitrate (NO_3_-N), total phosphorous (TP), phosphate (PO_4_-P), total sulphur (TS), sulphate (SO_4_-S), calcium (Ca) and total potassium (TK) values were determined using standard methods [22].

CH_4_ emissions from the plots were measured once a week in the following month after the fertilisation and, henceforth, in two-week intervals throughout the vegetation periods (9 times per year) in 2012 and 2014, using a closed static chamber method (three chambers per plot), as described by Järveoja *et al*. [23]. All the CH_4_ measurements were performed between 10 a.m. and 3 p.m. CH_4_ concentrations in the collected air were determined using the Shimadzu GC-2014 gas-chromatographic system (ECD, FID) combined with a Loftfield autosampler [24]. Peat temperature (at depths of 10, 30 and 40 cm) and groundwater table (WT) depth were measured on plots at each gas measurement campaign.

The study was carried out on private lands and the owner (Tootsi Turvas AS) of the lands gave permission to conduct the study on these sites.

### DNA extraction and quantitative PCR

DNA was extracted using a PowerSoil^®^ DNA Isolation kit (Mobio Laboratories Inc., Carlsbad, CA, USA) according to the manufacturer’s instructions. The homogenisation treatment was performed at 5,000 rpm for 20 s using Precellys^®^ 24 (Bertin Technologies, France). The extracted DNA was stored at −20°C until used in downstream analyses. The quantity and quality of the extracted DNA were determined using spectrophotometry (Infinite M200, Tecan AG, Austria).

Quantitative PCR (qPCR) was applied for the quantification of bacterial and archaeal 16S rRNA genes and methanogenic archaeal marker gene *mcrA* (S1 Table). New primer pairs were designed for the amplification of archaeal 16S rRNA gene and *mcrA* gene fragments. Details of the qPCR method are given in S1 Methods.

### Statistical analyses

In further data analyses, soils were grouped according to their management type as follows: uncultivated control (UC) and uncultivated fertilised soils (UF), and *Phalaris* cultivated controls (PC) and *Phalaris* cultivated fertilised soils (PF). The Between-Class Analysis (BCA) was applied in order to find the principal components based on the centre of gravity of log-transformed values of soil chemical parameters using a single factor (sampling time, soil layers, cultivation or cultivation and fertilisation) as instrumental variable. In addition, independent t-test, one-way ANOVA and Tukey HSD post hoc tests were applied to evaluate the significance of the differences between groups in physicochemical parameters and emission values according to the instrumental variable. Furthermore, an independent t-test was used to determine differences in chemical and gene parameter values as well as in soil temperature, WT and CH_4_ emission, between control and treatment plots, before fertilisation in 2012. An independent t-test was also used to determine differences in means of vegetation periods’ soil temperature, WT and CH_4_ between studied groups after the fertilisation in 2012. Spearman’s Rank correlation coefficient was applied in order to determine significant relationships between different gene parameter values, as well as between gene parameters and means of soil temperature, WT and CH_4_ emission values of vegetation periods in the studied soil groups.

A powerful technique for analysing ecological data is the usage of linear mixed-effects models (LMM) that enable simultaneous consideration of all the factors that potentially contribute to the understanding of the structure of the data [25]. For each of the studied soil layers and the studied gene parameters, a separate LMM was applied to test gene parameter relationships in cases of different grouping factors and with soil chemical variables. Detailed descriptions of applied data analysis methods are given in S2 Methods.

## Results

### Peat physicochemical parameters

The WT was from 6 to 67 cm below the surface during the studied vegetation periods and varied between plots within soil groups and also between study years (Table 1). In general, the temperature decreased towards the soil bottom layer in all sampling occasions and was the highest in the 0–20 cm layer, compared to the 20–40 and 40–60 cm layers (ANOVA, p<0.001 in both cases) (Table 1). Before the first fertilisation occasion in June 2012, there were no differences between PC and PF as well as between UC and UF in all measured soil physicochemical parameters values. In all plots, the soil temperature was significantly lower in 2012 than in 2014 (t-test, p<0.001). In 2012, the WT was significantly higher in cultivated soils than in uncultivated soils (t-test, p<0.001). Moreover, the WT was higher also in PC and PF in 2012 than in 2014 (t-test, p<0.01 and p<0.001, respectively).

**Table 1:** Mean values and standard deviations (in parentheses) of soil physical parameters and methane emissions (sampling time values in June 2012 (J12, n = 3) and study years vegetation periods values for 2012 and 2014 (S12 and S14, respectively; n = 9 in both cases)) for four studied soil groups (SG).

SG	ST	Soil physical parameters				CH_4_ emission (μg CH_4_-C m^–2^ h^–1^)
		WT (cm)	Temperature (°C)			
			10 cm	30 cm	40 cm	
UC	J12	−53 (6)	13.8 (0.3)	12.3 (0.3)	11.4 (0.4)	4.07 (2.15)
	S12	−41 (7)	14.3 (1.8)	13.6 (0.9)	12.9 (0.8)	8.25 (8.44)
	S14	−43 (6)	16.9 (3.9)	14.4 (1.8)	13.9 (1.5)	4.55 (2.54)
UF	J12^a^	−65 (2)	13.8 (0.3)	12.3 (0.3)	11.4 (0.4)	4.05 (2.36)
	S12	−46 (13)	14.3 (1.9)	13.7 (1.0)	13.0 (0.9)	6.52 (2.69)
	S14	−47 (8)	17.1 (3.9)	14.6 (2.0)	14.2 (1.7)	6.32 (2.13)
PC	J12	−50 (10)	13.8 (1.9)	12.2 (0.6)	11.5 (0.5)	7.80 (9.97)
	S12	−24 (11)	14.8 (1.7)	14.2 (1.0)	13.3 (0.9)	7.46 (6.97)
	S14	−42 (14)	16.7 (3.7)	14.7 (1.8)	14.2 (1.7)	0.62 (2.24)
PF	J12^a^	−48 (3)	13.8 (1.9)	12.2 (0.6)	11.5 (0.5)	8.36 (10.66)
	S12	−21 (12)	15.0 (1.9)	14.4 (1.3)	13.6 (1.4)	18.49 (11.93)
	S14	−43 (17)	17.2 (3.9)	15.1 (1.8)	14.8 (1.7)	0.99 (1.91)

UC, uncultivated control soils; UF, uncultivated fertilised soils; PC, *Phalaris* cultivated control soils; PF, *Phalaris* cultivated fertilised soils; ST, sampling time; WT, groundwater table.

^a^ Before the first fertilisation occasion.

**Fig 1:**
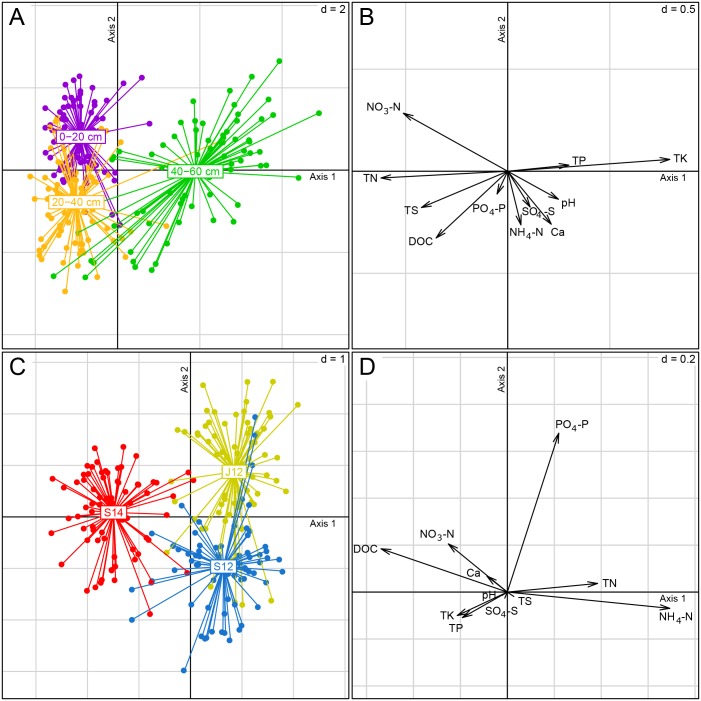
Between-group analysis (BCA) on the chemical parameters with the layers (A, B) or sampling times (C, D) as explanatory variable. Axis 1 of the BCAs accounted for 31.1% and axis 2 for 21.0% of the overall data variation, and 100% of projected variation. Abbreviations: J12 –June 2012, S12 –September 2012, S14 –September 2014, DOC—dissolved organic carbon, TN—total nitrogen, TP—total phosphorous, TS—total sulphur, TK—total potassium.

Soil chemical composition varied between soil layers but, in addition, the difference was also detected in the same layer of a plot at different sampling times (S2 Table). The BCAs using soil layer or sampling time as a grouping factor described 20.1% and 15.3% of the total soil chemical parameters variation, respectively (Monte Carlo test, p<0.001). According to BCA results, peat chemical composition of two upper layers differed mainly by NH_4_-N levels (Fig 1). The 40–60 cm layer was characterised by higher TK, TP and pH values, and lower TN, NO_3_-N and TS values compared to the upper layers. In the case of the sampling occasions, the main difference was allocated to the difference between year 2012 and 2014. Year 2012 samples had higher NH_4_-N and TN concentrations and smaller DOC values compared to year 2014. Two sampling occasions of the year 2012 were separated by PO_4_-P content that was higher in June samples.

Although cultivation, as well as cultivation and fertilisation, explained less than 3.6% of the total variation in peat chemical composition (Monte Carlo test, p<0.001 and p<0.05, respectively), several significant differences between studied soil groups were detected. Cultivated plots had significantly lower pH than uncultivated plots (t-test, p<0.05). Similarly, the content of NO_3_-N in soil differed between uncultivated and cultivated soils (t-test, p<0.001). UF had significantly higher NO_3_-N content than UC (t-test, p<0.01). In addition, PO_4_-P and Ca concentrations were both lower in uncultivated soils than in cultivated soils (t-test, p<0.001 in both cases). The content of PO_4_-P was higher in UF than in UC (t-test, p<0.05).

### Abundance of target genes and relationships between gene parameters

**Fig 2:**
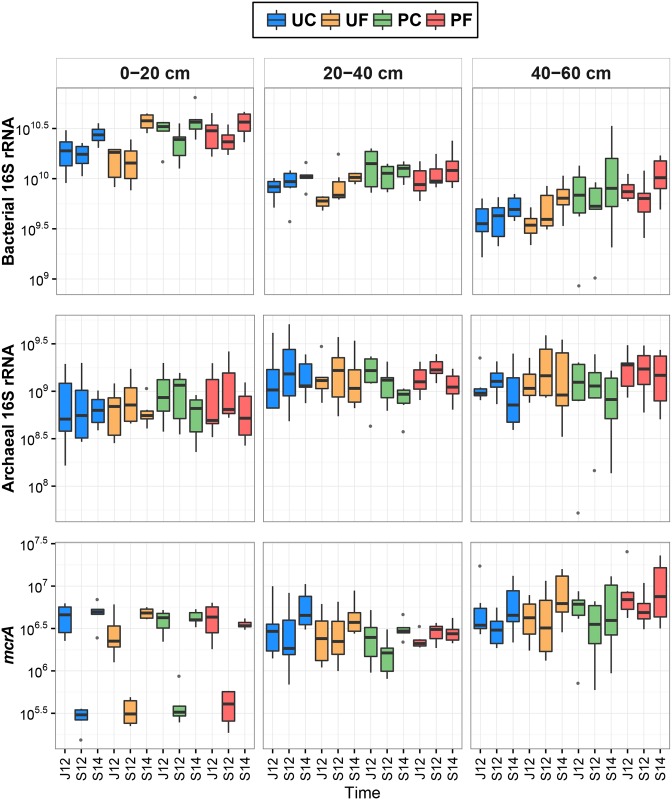
Box plots of target gene copy numbers (copies/g dry weight) in four studied soil groups of three depths collected in June 2012 (J12), September 2012 (S12), and September 2014 (S14). The central line is the median, the edges of the box are the 25th and 75th percentiles, the whiskers represent the 95% confidence interval, and grey dots indicate outliers. Abbreviations: UC—uncultivated control soils, UF—uncultivated fertilised soils, PC–*Phalaris* cultivated control soils, PF–*Phalaris* cultivated fertilised soils.

The bacterial 16S rRNA gene copy numbers ranged from 8.6×10^8^ to 6.5×10^10^ gene copy numbers per gram of dry weight (copies/g dw) across all the studied samples (Fig 2). Significantly higher values of this parameter were measured in all cultivated soils, compared to the uncultivated soils (t-test, p<0.05). Statistical analyses confirmed that there were not significant differences between PC and PF as well as between UC and UF in this gene abundance before fertilisation in June 2012. There was also no difference between soil layers of differently treated plots, according to LMM (S3 Table). In general, the bacterial 16S rRNA gene decreased with depth: significantly higher values were measured in the 0–20 cm layer compared to the two lower layers (ANOVA, p<0.001 in both cases) and in the 20–40 cm layer compared to the 40–60 cm layer (ANOVA, p<0.01). The abundance of this gene was lower in 2012 (both June and September samples) than in September 2014 (ANOVA, p<0.01 and p<0.001, respectively).

The archaeal 16S rRNA gene abundance ranged from 5.3×10^7^ to 5.1×10^9^ copies/g dw across all the studied peat samples (Fig 2), and there was no difference in abundance between uncultivated and cultivated soils. The statistical analyses also showed no difference between PC and PF, as well as between UC and UF in this gene abundance before fertilisation in June 2012. This parameter was higher in PF than in PC (t-test, p<0.05), and the difference was especially pronounced in the 40–60 cm layer (S3 Table). In general, the archaeal gene abundance was smaller in the 0–20 cm soil layer than in two lower layers (ANOVA, p<0.001 in both cases) and was significantly higher in September 2012 than in September 2014 (ANOVA, p<0.05).

**Fig 3:**
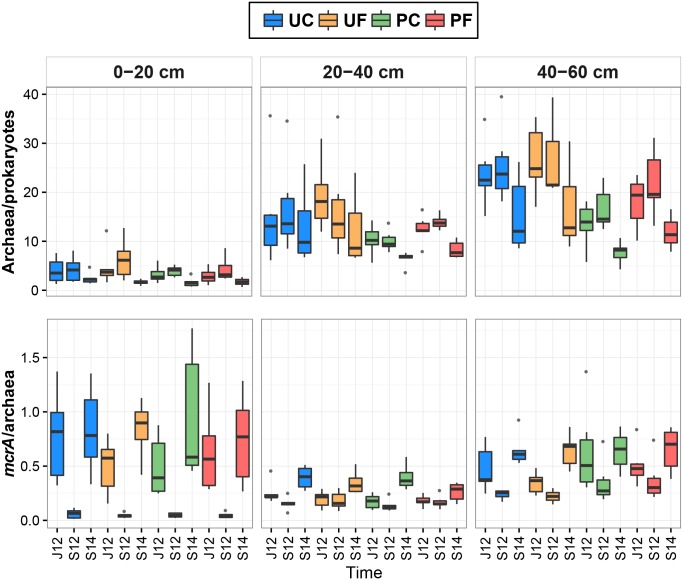
Box plots of archaea proportion in prokaryotes and *mcrA* proportion in archaea (%) in four studied soil groups of three depths collected in June 2012 (J12), September 2012 (S12), and September 2014 (S14). The central line is the median, the edges of the box are the 25th and 75th percentiles, the whiskers represent the 95% confidence interval, and grey dots indicate outliers. Abbreviations: UC—uncultivated control soils, UF—uncultivated fertilised soils, PC–*Phalaris* cultivated control soils, PF–*Phalaris* cultivated fertilised soils.

The proportion of archaea in the prokaryotic community ranged from 0.67 to 39.56% across all the samples (Fig 3). The archaeal proportion was significantly higher in all uncultivated soils compared to the cultivated soils (t-test, p<0.001). The proportion of archaea was not different between the control and the fertilised plots soils (UC vs. UF and PC vs. PF) before fertilisation in June 2012. The archaeal proportion was significantly higher in the 40–60 cm layer of PF than in the respective PC layer (S3 Table). In general, the 0–20 cm layer community had a smaller archaeal proportion than the two lower layers (ANOVA, p<0.001 in both cases) and the 20–40 cm layer had a smaller archaeal proportion than the 40–60 cm layer (ANOVA, p<0.001). These parameter values were higher in 2012 (both June and September) samples than in September 2014 samples (ANOVA, p<0.01 and p<0.001, respectively).

The *mcrA* abundance ranged from 1.6×10^5^ to 2.6×10^7^ copies/g dw across all the samples (Fig 2), and there was no difference in abundance between uncultivated and cultivated soils. Similarly to the other gene parameters, these parameter values were not significantly different in control and fertilised plots (UC vs. UF and PC vs. PF) before fertilisation in June 2012. The *mcrA* abundance was higher in the 40–60 cm peat layer of PF compared to the respective layer of PC (S3 Table). In general, the *mcrA* abundance values were smaller in the 0–20 and 20–40 cm layers than in the bottom soil layer (ANOVA, p<0.001 in both cases). The abundance was lower in September 2012 in comparison with June 2012 (ANOVA, p<0.01) and September 2014 (ANOVA, p<0.001).

The *mcrA* proportion ranged from 0.01 to 1.77% in the archaeal communities of all the studied soil samples (Fig 3), and there was no difference in proportion between uncultivated and cultivated soils. Statistical analyses also did not detect any differences between PC and PF as well as between UC and UF before fertilisation in June 2012. Furthermore, no differences were detected between soil layers of differently treated plots by LMM (S3 Table). In general, this proportion was higher in the 0–20 and 40–60 cm layers than in the 20–40 cm layer (ANOVA, p<0.001 in both cases). Similarly to the *mcrA* abundance, this gene proportion was also lower in September 2012 than in June 2012 and September 2014 (ANOVA, p<0.001 in both cases). In addition, *mcrA* proportion was lower in June 2012 soils than in soils collected in September 2014 (ANOVA, p<0.001).

A number of significant relationships between the studied genes parameters were obtained but the pattern of the relationships varied between the studied soil groups (S4 Table). There were positive correlations between the archaeal and bacterial 16S rRNA genes abundances that were revealed either in the 20–40 cm layer (PF) or in the 40–60 cm layer (UF) or in both soil layers simultaneously (PC). Strong positive correlations were found between bacterial 16S rRNA gene and *mcrA* abundances in both uncultivated and cultivated soil groups either in the 40–60 cm layer (UC) or in the 0–20 and 40–60 cm layers simultaneously (UF and PC). The *mcrA* abundance was related to the archaeal 16S rRNA gene abundance in the 20–40 cm layer of both uncultivated soil groups and also in the 40–60 cm layer of all soil groups. The *mcrA* abundance was related to the archaeal 16S rRNA gene proportion in the 0–20 cm layer of UF and, in addition, in both cultivated soil groups, while in the 20–40 cm soil layer the revealed relationship with proportion (UF) was positive.

### Relationships between target genes and soil physicochemical parameters

The Spearman’s Rank correlation coefficient was used to determine relationships between gene parameters (measurements of September 2012 and 2014) and vegetation periods`soil physical parameters (S4 Table). In control plots (UC and PC), bacterial 16S rRNA gene abundance was positively related to the soil temperature in the 0–20 cm layer of the peat. Separate LMM was assessed for each of the studied gene parameters and soil layers (S3 Table). The bacterial 16S rRNA gene abundance was related to soil pH in the 0–20 and 40–60 cm layer. This gene parameter was also related to DOC concentration in the 20–40 cm layer and TP content in the 40–60 cm layer of residual peat.

Statistical analyses found a positive relationship between archaeal 16S rRNA gene abundance and WT in the 20–40 cm soil layer of UC. The abundance of archaeal 16S rRNA gene was correlated to TS, SO_4_-S and Ca content in the 0–20 cm soil layer and to TP content in the 20–40 and 40–60 cm layers. In addition, this parameter was related to PO_4_-P concentration in the 40–60 cm layer.

Statistical analyses detected relationships between archaeal proportion and soil temperature as well as WT in different peat layers. The archaeal proportion in the prokaryotic community was correlated to TS content in all of the studied peat layers. Additionally, the relationships between this parameter and DOC, NH_4_-N, SO_4_-S, TK and Ca content in the 0–20 cm layer, and relationship with TP in the 20–40 cm layer were revealed. In the 40–60 cm layer, correlations between archaeal proportion and soil TN and PO_4_-P content were found.

Strong positive correlations between abundance of *mcrA* and soil temperature were revealed in the 0–20 and 20–40 cm layers of PC and in the 40–60 cm layer of UC, while negative correlations were observed between the abundance of *mcrA* and WT in the 0–20 cm layer of PC and PF. The abundance of *mcrA* was significantly related to TN and NH_4_-N content in the 0–20 cm layer, and to pH and NO_3_-N content in the 20–40 cm peat layer. Furthermore, a relationship between *mcrA* abundance and DOC was detected in the 20–40 and 40–60 cm layers and, additionally, a relationship with TP was found in the 40–60 cm layer.

The proportion of *mcrA* in the archaeal community was positively related to soil temperature (mainly in the 40–60 cm layer) and negatively related to the WT in cultivated soils. In addition, this parameter was significantly related to TS content in the 0–20 cm layer, and to PO_4_-P content in the 40–60 cm peat layer.

### Methane emission and relationships between gene parameters and methane emission

The average CH_4_ emission for the vegetation periods was −4.6 to 28.8 μg CH_4_-C m^–2^ h^–1^ from the studied plots (Table 1). The CH_4_ emission from uncultivated and cultivated plots was not different. A considerably higher emission was detected from cultivated soils in 2012 than in 2014 (t-test, p<0.05). Statistical analysis revealed a strong negative correlation between the bacterial 16S rRNA gene abundance in the 0–20 cm layer and the CH_4_ emission in cultivated soils (S4 Table). Although the archaeal 16S rRNA gene abundance was not related to the CH_4_ emission in any of the studied soils, a positive relationship between CH_4_ emission and the proportion of archaea in the prokaryotic community in the 40–60 cm layer of PF was revealed. The *mcrA* abundance in deeper layers of uncultivated peat was positively related to the CH_4_ emission. Negative relationship was found between *mcrA* abundance in the 0–20 cm layer of cultivated peat and CH_4_ emission, and the cause of this relationship needs further investigation. The analyses did not detect any significant relationships between *mcrA* proportion in the archaeal community and CH_4_ emission for the studied soil groups.

## Discussion

### Methane emissions and environmental conditions in residual peat column

In this study, we focused on the response of the soil microbial abundance to the reclamation of an abandoned peat extraction area with a bioenergy crop in order to understand the effect of actions related to this peatland management type on peat properties, as well as prokaryotic abundance and methanogenic potential. The CH_4_ emission rates were relatively small and in many cases even some consumption of this gaseous compound was detected from the peat of the study plots, although the box measurements might underestimate daily methane emissions as has been shown by a study comparing box and tower measurements results in a boreal wetland [26]. The emission from the plots stayed in the range (−26 to 66 μg CH_4_-C m^–2^ h^–1^) of those reported previously in different disturbed peatlands: semidry pine-dominated mesotrophic peatland forest [27]; drained and hay cultivated peatlands [28]; abandoned peat extraction areas and its different reed canary grass treatments [12]. In contrast, a previous study found much higher and more variable CH_4_ emissions (median 654 μg CH_4_-C m^–2^ h^–1^ and range −2 to 12,915 μg CH_4_-C m^–2^ h^–1^, respectively) from the Lavassaare natural peatland area during two vegetation periods. These differences between disturbed and natural sites in CH_4_ fluxes can be attributed to the different substrate quality as well as the lowered groundwater level and concordant changes in redox conditions in the peat column that inhibit the activity of methanogens and enhance CH_4_ oxidising methanotrophs [10,29].

Our results indicate that climate conditions had a strong effect on soil physiochemical characteristics and caused most of the temporal differences that were detected in two study years. Notably, a higher water table was recorded in cultivated soils during the vegetation period of the wetter year (2012), whereas in the dryer year this difference was not detectable. The difference between water statuses in these two treatment plots can be attributed to the peculiarities in groundwater dynamics at different areas of the study site [23].

### Effect of reed canary grass cultivation and fertilisation on prokaryotic community abundance and CH_4_ emission in residual peat

Our study further support that reed canary grass cultivation mitigated CH_4_ emission [18], however, methanogen abundance remained approximately the same or even increased in different layers of residual peat under cultivated sites over time. This fact may indicate that methanogens were not very active microorganisms in cultivated peat or methanotrophic bacteria, supported by the plant exudates, consumed most of the CH_4_. Furthermore, the increased bacterial abundance in the top layer of cultivated peat probably caused a consumption of CH_4_ which was produced at lower anaerobic sites and led to the decrease in CH_4_ emission from these soils [20]. As indicated by the positive relationships between *mcrA* abundance and gas emission in uncultivated peat, the two lower layers influenced most the CH_4_ emission while in cultivated peat such types of relationships were missing. Juottonen *et al*. [30] showed a similar relationship in the anaerobic (7.5–15 cm) layer of restored forestry drained boreal peatlands.

Fertilisation had little influence on CH_4_ emission, and a few relationships between the emission and gene parameters in abandoned peat extraction area were found. Nevertheless, several studies have shown that inorganic fertilisers inhibit methanotrophs while stimulating methanogens in various environments such as rice paddies, grassland and forests (reviewed by Nazaries *et al*. [20]). Moreover, the addition of mineral N may alleviate N-limitation for methanogens [31,32], but there can be an opposite effect in different ecosystems. Kim *et al*. [33] showed that combined N and P addition decreased CH_4_ production in the top layer (0–5 cm) of drainage ditch sediment due to the increased substrate competition with denitrifiers. A large part of the uncertainty in the effect of nitrogen on methane emission can probably address the varying plant responses which depend on plant species, environmental conditions, and on the type and amount of fertiliser as was shown in agricultural wetlands [34].

### Effect of reed canary grass cultivation and fertilisation on residual peat properties and prokaryotic community abundance

Our results indicate that reed canary grass cultivation on abandoned peat extraction area had an effect on peat chemical properties: lowered pH and NO_3_-N content as well as increased PO_4_-P and Ca content. Philippot *et al*. [35] showed that the uptake of nutrients and secretion of exudates by plant roots affects soil pH and nutrient availability in soil. Hence, Andersen *et al*. [36] showed that the presence of plants can increase the efficiency of the maintenance of nutrients in the plant-soil system and prevent nutrients loss from the residual peat by leaching. We observed the increase in amounts of soluble fractions of P and N (NO_3_-N) as a response of mineral fertilisation only in uncultivated peat.

The results of this study showed an increase in the bacterial community abundance in response to the reed canary grass cultivation, while the addition of nutrients did not have an effect on bacterial abundance. Tavi *et al*. [37] showed a significant increase in microbial biomass and activity in the surface layer (0–15 cm) of reed canary grass-cultivated cutover peatland compared to the bare peat site. The carbon input from plants may support bacterial communities that have a functionally distinct role in carbon turnover, as was shown for the fungal communities in abandoned cutover peatlands [10]. Hence, the archaeal proportion in the prokaryotic community was higher in uncultivated soils; the archaeal abundance was similar in cultivated and uncultivated peat. As a result of mineral fertiliser application, we observed an increase in archaeal abundance, especially in the deepest layer of cultivated peat.

Notwithstanding, in general, we did not find any differences in methanogen abundances of respective layers between cultivated and uncultivated soils. Niu *et al*. [38] found also that cultivation did not have a discernible effect on the abundance of methanogens in constructed wetlands. The effect of fertilisation was revealed in the 40–60 cm layer of fertilised cultivated peat where these organisms were more abundant, compared to the same layer of the unfertilised cultivated peat. This result may be explained by the fact that fertilisation boosted plant growth and carbon from plant roots and anoxic conditions probably provided favourable conditions for methanogens in this layer. The results of this study indicate that the addition of mineral fertiliser affected microbial relationships in uncultivated plots, while several relationships emerged between gene parameters after fertilisation.

### General trends in prokaryotic community abundance in residual peat column

The results of the current study indicate that bacteria dominated over archaea throughout the residual peat column. Similarly to the vertical trends in soil chemical properties, we found systematic changes from the top to the bottom layer in abundance of different microbial groups in all the studied plots. The bacterial abundance decreased from the top to the bottom layer, whereas the archaeal abundance and proportion in the prokaryotic community had an opposite vertical gradient direction. The archaeal proportion in the prokaryotic community in the top peat layer stayed in the range of the previously reported proportions (in most cases up to 5%, occasionally up to 10%) found in the 5–10 cm layer of various terrestrial habitats (forests, grasslands, permafrost areas, lake sediments, etc.) all over the world [39,40], but in two deeper layers it was substantially higher. The relative abundance of methanogens in residual peat was the smallest in the middle of the peat column and the largest potential for methanogenesis was found in the bottom peat layer. The vertical stratification of microbial communities is reported in all types of natural peatland and is attributed to the carbon quality and energy constraints—variation in combination of available O_2_ and other electron acceptors with depth in peat [10].

A systematic decrease in methanogens and their proportion in the archaeal community were detectable in the upper 20 cm layer of the studied peat during the study period. Mäkiranta *et al*. [41] suggest that as a result of peat mining, the originally deeper layers in the peat column contained peat of several thousand years in age which appears at the surface where the methanogenic community has to adapt to the new conditions prevailing in residual peat. Hence, as anaerobic organisms, methanogens are shown to be flexible: they tolerate energy stress, and by relocating in anaerobic micro-habitats found in unfavourable environments, they survive and even remain active [10].

Our results indicate that the soil physical parameters affecting the abundance of different microbial groups varied between soil layers. The groundwater level in the peat significantly affected the archaeal community abundance and structure (proportion of methanogens in the community), although we did not detect a similar effect on bacterial community abundance.

In residual peat, strong relationships between soil chemical factors and microbial community abundance/proportion were revealed, but the relationship types varied between layers. The obtained results refer to various processes taking place at different depths of the peat column. In addition to the already well-known factors (i.e. pH, DOC, different fractions of N and P) affecting soil microbial abundance, our results indicate importance of total sulphur pool and its dissolved fraction in case of archaeal abundance and proportion in the prokaryotic community. In soils, archaea participate in various sulfidogenic and sulphide oxidation processes [16]. In the two deeper layers, methanogen abundance was linked to the amount of DOC that can be an important carbon source for methanogenesis in northern peatland soils [42]. The size of the total P pool, as well as its soluble fraction, had an effect on methanogen abundance and proportion in residual peat. Phosphorous is an important factor regulating methanogenesis in P-limited environments. Medvedeff *et al*. [43] concluded from the results of their study, that the addition of P increased the activity of methanogens either directly or indirectly via fermentative bacteria that produce methanogenic substrates in P-limited calcareous subtropical wetland soil.

## Conclusions

In general, the methanogen abundance was low in the archaeal community, but the highest CH_4_ production potential was revealed at the bottom of the peat column at abandoned peat extraction area. In response to the reed canary grass cultivation, the physicochemical status of peat was slightly changed and bacterial abundance increased. In uncultivated peat, the two deeper layers influenced mainly the CH_4_ emission, while in cultivated peat, the more pronounced effect of methanotrophic bacteria on CH_4_ emission can be assumed from the obtained results. Overall, reed canary grass cultivation reduced CH_4_ emission, although methanogen abundance remained approximately the same or even increased in different layers of residual peat under cultivated sites over time. The results of the current study indicate that the conversion of abandoned peat extraction areas into reed canary grass cultivations mitigate methane emission from peat due to associate changes in peat microbial community abundance.

## Supporting Information

S1 Methods Quantitative PCR. (DOCX)

S2 Methods Statistical analyses. (DOCX)

S1 Table The characteristics of the qPCR primer sets and programmes. (DOCX)

S2 Table Mean values and standard deviations of soil chemical parameters in different peat layers of the studied soil groups at the sampling times. (DOCX)

S3 Table Statistically significant relationships between different gene parameters and soil chemical variables, and between gene parameters of different treatments in three layers. (DOCX)

S4 Table Statistically significant relationships between different gene parameters, and between gene parameters and means of the study years vegetation periods`soil physical parameters as well as methane emissions in three layers of the studied soil groups. (DOCX)
