# Supplementary material for: Impact of Reed Canary Grass Cultivation and Mineral Fertilisation on the Microbial Abundance and Genetic Potential for Methane Production in Residual Peat of an Abandoned Peat Extraction Area
Source: PLoS One. 2016 Sep 29;11(9):e0163864. doi: 10.1371/journal.pone.0163864 (PMC5042519; doi:10.1371/journal.pone.0163864)
Supplement: S1 Methods — (DOCX) [file pone.0163864.s001.docx]

**S1 Methods. Quantitative PCR**

Quantitative PCR (qPCR) was applied for the quantification of bacterial and archaeal 16S rRNA genes and methanogenic archaeal marker gene *mcrA*. New primer sets were designed for the amplification of archaeal 16S rRNA gene and *mcrA* gene fragments (S1 Table). These primers were designed on the basis of the multiple sequence alignments of full-length gene sequences from National Center for Biotechnology Information (NCBI) database and checked for melting temperatures, potential primer-dimer and hairpin structures. Primers with high coverage of the target group and specificity according to The Ribosomal Database Project (RDP) database for archaeal 16S rRNA gene or Functional Gene Pipeline/Repository (FGPR) database for *mcrA* gene were used successfully in empirical testing on various types of environmental samples (peat materials of different decomposition degree, agricultural soils, filter materials of constructed wetlands).

The qPCR assays were performed using RotorGene^®^ Q (Qiagen, CA, USA). Stock solution of target sequence containing plasmid (Eurofins MWG Operon, Germany) was used to create a serially diluted standard curve for bacterial 16S rRNA, ranging from 10^4^ to 10^8^ copies. The optimised qPCR reactions were performed in 10 μl volume containing 5 μl Maxima SYBR Green Master Mix (Thermo Fisher Scientific Inc., MA, USA), an optimised concentration of forward and reverse primers (S1 Table), 1 μl of template DNA, and sterile distilled water. The optimised reaction conditions for each target gene are described in S1 Table. All qPCR measurements were performed in triplicates and negative controls were included in each qPCR run.

For qPCR data analyses Rotor-Gene Series software, version 2.0.2 (Qiagen), the LinRegPCR program, version 2013.0 [1] were used. The recorded amplification efficiencies for bacterial 16S rRNA gene standard dilutions as well as for the soil samples` *mcrA* and bacterial and archaeal 16S rRNA genes were the following: 1.864±0.034, 1.464±0.087, 1.852±0.037 and 1.808±0.053, respectively. In order to calculate the abundance of bacterial 16S rRNA gene in soil sample an estimation of a fold difference (FD) between a sample (A) and each 10-fold standard dilution (B) was calculated using formula (1) proposed by Ruijter et al. [1] as follows:

FD = N_0,A_ / N_0,B_ = (N_t,A_ / E_A_^Ct,A^) / (N_t,B_ / E_B_^Ct,B^), (1);

where N_0_ stands for the starting concentrations of the A and B amplicons (in arbitrary fluorescence units), E for amplification efficiencies, N_t_ for fluorescence threshold values, and C_t_ for the fractional number of cycles needed to reach the fluorescence threshold. The estimates of gene copy numbers in samples were calculated by multiplying each obtained FD value with the known number of gene copies in the respective standard dilution. The final gene abundance in each sample was calculated as a mean of all obtained abundance estimates and presented as gene copy numbers per gram of dry weight (copies/g dw).

The proportion calculations were performed using the calculation formula (1) based in each case on FD value of the compared genes (amplicons A and B) used in normalisation. The proportion of archaea in microbial community was calculated by normalising archaeal 16S rRNA genes against a sum of bacterial 16S rRNA and archaeal 16S rRNA genes. The proportion of methanogenic organisms in archaeal community was calculated by normalising *mcrA* genes against archaeal 16S rRNA genes. Abundances of archaeal 16S rRNA and *mcrA* genes were calculated as relative abundances from the abundance of the reference gene (bacterial 16S rRNA).

**References**

1. Ruijter JM, Ramakers C, Hoogaars WMH, Karlen Y, Bakker O, van den Hoff MJB, et al. Amplification efficiency: Linking baseline and bias in the analysis of quantitative PCR data. Nucleic Acids Res. 2009;37: e45. doi:10.1093/nar/gkp045
