# Supplementary material for: Impact of Reed Canary Grass Cultivation and Mineral Fertilisation on the Microbial Abundance and Genetic Potential for Methane Production in Residual Peat of an Abandoned Peat Extraction Area
Source: PLoS One. 2016 Sep 29;11(9):e0163864. doi: 10.1371/journal.pone.0163864 (PMC5042519; doi:10.1371/journal.pone.0163864)
Supplement: S2 Methods — (DOCX) [file pone.0163864.s002.docx]

**S2 Methods. Statistical analyses**

In data analyses, soils were grouped according to their management type as follows: uncultivated control (UC) and uncultivated fertilised soils (UF), and *Phalaris* cultivated controls (PC) and *Phalaris* cultivated fertilised soils (PF). The Between-Class Analysis (BCA) was applied in order to find the principal components based on the centre of gravity of log-transformed values of soil chemical parameters using a single factor (sampling time, soil layers, cultivation or cultivation and fertilisation) as instrumental variable. The significance of the differences between analysed groups was tested using a Monte-Carlo permutation test (9,999 permutations). The BCA was performed using the ade4 package version 1.7-4 [1] and the software R version 3.3.0 [2]. In addition, independent t-test, and one-way ANOVA and Tukey HSD post hoc tests were applied to evaluate the significance of the differences between groups in physicochemical parameters and emission values according to the instrumental variable. The differences between soil groups at three soil layers were analysed using the STATISTICA 7.1 (StatSoft, Inc.).

Additionally, an independent t-test was also used to determine differences in chemical and gene parameter values as well as in soil temperature, groundwater depth (WT) and CH_4_ emission, between control and treatment plots, before the fertilisation in 2012. An independent t-test was also used to determine differences in means of vegetation periods’ soil temperature, WT and CH_4_ between studied groups after the fertilisation in 2012. Spearman’s Rank correlation coefficient was applied in order to determine significant relationships between different gene parameter values (measurements of September 2012 and 2014), as well as between gene parameters (measurements of September 2012 and 2014) and means of soil temperature, WT and CH_4_ emission values of vegetation periods in the studied soil groups.

For each of the studied soil layers and the studied gene parameters, a separate linear mixed-effects model (LMM) was applied to test gene parameter relationships in cases of different grouping factors and with soil chemical variables using the “lmer” method (version 1.1-6) [3] while p-values were calculated in order to confirm the significance of the relationships using the lmerTest package version 2.0-3 [4] in R version 3.3.0 [2]. Data obtained from control plots at each sampling time were included into the models whereas in the cases of fertilised plots the June 2012 (before fertilisation) data were excluded. In the cases of gene abundance and proportion variables, logarithmic and arcsine square root transformations, respectively, were used to fulfil the model requirements. The fitted LMMs were mixed effects models containing both fixed and random effects. As fixed effects, categorical explanatory variables were the presence or absence of vegetation (uncultivated/cultivated) and their interaction with fertilisation (unfertilised/fertilised); continuous explanatory variables were soil chemical parameters. The crossed random effects (sampling points distance and sampling time) on the variability of gene parameters were also taken into account in the models. Obvious deviations from normality or homoscedasticity were not observed in the residual plots. None of the checks indicated any severe violation of the assumptions.

In all cases, statistical significance was determined at the 95% confidence level.

**References**

1. Dray S, Dufour AB. The ade4 Package: Implementing the Duality Diagram for Ecologists. J Stat Softw. 2007;22: 1 – 20. doi:10.1.1.177.8850

2. R Development Core Team. R: A language and environment for statistical computing [Internet]. Vienna: R Foundation for Statistical Computing; 2016. Available: https://www.r-project.org/

3. Bates D, Mächler M, Bolker B, Walker S. lme4: Linear mixed-effects models using Eigen and S4 [Internet]. 2014. Available: http://cran.r-project.org/package=lme4

4. Kuznetsova A, Brockhoff PB, Bojesen Christensen RH. lmerTest: Tests for random and fixed effects for linear mixed effect models (lmer objects of lme4 package) [Internet]. 2013. Available: http://cran.r-project.org/package=lmerTest
