# Supplementary material for: Impact of Reed Canary Grass Cultivation and Mineral Fertilisation on the Microbial Abundance and Genetic Potential for Methane Production in Residual Peat of an Abandoned Peat Extraction Area
Source: PLoS One. 2016 Sep 29;11(9):e0163864. doi: 10.1371/journal.pone.0163864 (PMC5042519; doi:10.1371/journal.pone.0163864)
Supplement: S1 Table — (DOCX) [file pone.0163864.s003.docx]

**S1 Table.** **The characteristics of the qPCR primer sets and programmes.**

| Primer | Sequence (5′-3′) | Target gene | Amplicon size (bp) | Primer con-centration (μM) | qPCR programme | Primer reference |
| --- | --- | --- | --- | --- | --- | --- |
| Bact517F | GCCAGCAGCCGCGGTAA | Bacterial 16S rRNA | 530 | 0.6 | 95°C 10 min; 35 cycles: 95°C 30 s; 60°C 45 s; 72°C 45s; Melting curve: 70-95°C | [1] |
| Bact1028R | CGACARCCATGCASCACCT^a^ |  |  |  |  | [2] |
| Arc519F | CAGYCGCCRCGGTAA^a^ | Archaeal 16S rRNA | 393 | 0.6 | 95°C 10 min; 45 cycles: 95°C 15 s; 56°C 30 s; 72°C 30s; Melting curve: 65-95°C | This study^b^ |
| Arch910R | GCYCCCCCGCCWATTC^a^ |  |  |  |  | This study^b^ |
| mcrA-F | CASTTYGGHGGWTCHCA^a^ | *mcrA* | 135 | 0.8 | 95°C 10 min; 55 cycles: 95°C 15 s; 51°C 30 s; 72°C 30s; Melting curve: 65-95°C | This study^b^ |
| mcrA-R | TGVAGRTCGTADCCRWAGAA^a^ |  |  |  |  | This study^b^ |

^a^ Degenerate base symbols: W = A/T; S = G/C; R = A/G; Y = C/T; D = A/G/T; H = A/C/T; V = A/C/G.

^b^ Targeting previously used conserved regions [3–6].

**References**

1. Liu Z, Lozupone C, Hamady M, Bushman FD, Knight R. Short pyrosequencing reads suffice for accurate microbial community analysis. Nucleic Acids Res. 2007;35: e120. doi:10.1093/nar/gkm541

2. Dethlefsen L, Huse S, Sogin ML, Relman DA. The Pervasive Effects of an Antibiotic on the Human Gut Microbiota, as Revealed by Deep 16S rRNA Sequencing. Eisen JA, editor. PLoS Biol. 2008;6: e280. doi:10.1371/journal.pbio.0060280

3. Giovannoni SJ, DeLong EF, Olsen GJ, Pace NR. Phylogenetic group-specific oligodeoxynucleotide probes for identification of single microbial cells. J Bacteriol. 1988;170: 720–726.

4. Großkopf R, Janssen PH, Liesack W. Diversity and Structure of the Methanogenic Community in Anoxic Rice Paddy Soil Microcosms as Examined by Cultivation and Direct 16S rRNA Gene Sequence Retrieval. 1998;64: 960–969.

5. Shigematsu T, Tang Y, Kobayashi T, Kawaguchi H, Morimura S, Kida K. Effect of dilution rate on metabolic pathway shift between aceticlastic and nonaceticlastic methanogenesis in chemostat cultivation. Appl Environ Microbiol. 2004;70: 4048–4052. doi:10.1128/AEM.70.7.4048-4052.2004

6. Colwell FS, Boyd S, Delwiche ME, Reed DW, Phelps TJ, Newby DT. Estimates of biogenic methane production rates in deep marine sediments at Hydrate Ridge, Cascadia margin. Appl Environ Microbiol. 2008;74: 3444–3452. doi:10.1128/AEM.02114-07
