# Supplementary material for: Impact of Reed Canary Grass Cultivation and Mineral Fertilisation on the Microbial Abundance and Genetic Potential for Methane Production in Residual Peat of an Abandoned Peat Extraction Area
Source: PLoS One. 2016 Sep 29;11(9):e0163864. doi: 10.1371/journal.pone.0163864 (PMC5042519; doi:10.1371/journal.pone.0163864)
Supplement: S2 Table — (DOCX) [file pone.0163864.s004.docx]

**S2** **Table. Mean values and standard deviations (in parentheses; n=6) of soil chemical parameters in different peat layers of the studied soil groups (SG) at the sampling times (ST).**

| **SG** | **ST** | **Layer (cm)** | **pHH_2_O** | **DOC**  **(mg/kg)** | **TN**  **(g/kg)** | **NH_4_-N**  **(mg/kg)** | **NO_3_-N**  **(mg/kg)** | **TP**  **(mg/kg)** | **PO_4_-P**  **(mg/kg)** | **TS**  **(g/kg)** | **SO_4_-S**  **(g/kg)** | **Ca**  **(mg/kg)** | **TK**  **(mg/kg)** |
| --- | --- | --- | --- | --- | --- | --- | --- | --- | --- | --- | --- | --- | --- |
| UC | J12 | 0–20 | 6.2 (0.6) | 207 (54) | 25.5 (3.3) | 6.8 (4.9) | 35.7 (10.7) | 260 (33) | 0.29 (0.19) | 8.8 (1.1) | 1.1 (1.3) | 481 (330) | 60 (19) |
|  |  | 20–40 | 6.3 (0.5) | 345 (113) | 27.2 (2.3) | 20.0 (14.1) | 15.5 (5.0) | 283 (42) | 1.12 (0.96) | 11.7 (3.3) | 2.1 (2.1) | 991 (702) | 111 (50) |
|  |  | 40–60 | 6.7 (0.4) | 184 (97) | 18.0 (6.5) | 11.1 (5.0) | 5.5 (5.4) | 313 (69) | 0.29 (0.17) | 5.6 (1.7) | 1.3 (1.2) | 623 (172) | 1,003 (664) |
|  | S12 | 0–20 | 6.1 (0.5) | 185 (25) | 25.3 (2.6) | 5.0 (4.3) | 46.6 (18.1) | 278 (25) | 0.07 (0.00) | 9.3 (1.1) | 1.2 (1.4) | 543 (422) | 76 (27) |
|  |  | 20–40 | 6.2 (0.5) | 267 (23) | 28.3 (2.1) | 18.1 (19.8) | 13.9 (11.7) | 288 (44) | 0.09 (0.02) | 11.6 (2.2) | 2.1 (2.5) | 810 (714) | 111 (99) |
|  |  | 40–60 | 6.5 (0.5) | 205 (109) | 18.4 (9.3) | 11.1 (11.2) | 7.5 (9.8) | 330 (43) | 0.08 (0.02) | 7.3 (2.3) | 1.6 (1.4) | 633 (373) | 760 (427) |
|  | S14 | 0–20 | 6.3 (0.6) | 487 (97) | 18.6 (6.3) | 1.1 (0.0) | 54.5 (10.8) | 268 (29) | 0.20 (0.03) | 8.9 (1.0) | 0.9 (0.9) | 495 (322) | 87 (34) |
|  |  | 20–40 | 6.3 (0.6) | 632 (200) | 19.3 (6.6) | 3.4 (2.0) | 21.2 (12.4) | 300 (46) | 0.28 (0.08) | 10.6 (2.5) | 1.7 (2.1) | 786 (703) | 295 (288) |
|  |  | 40–60 | 6.8 (0.7) | 386 (130) | 12.6 (5.0) | 3.1 (2.8) | 4.5 (2.8) | 342 (59) | 0.19 (0.05) | 6.9 (3.0) | 8.8 (9.6) | 610 (291) | 1,393 (474) |
| UF | J12^a^ | 0–20 | 6.1 (0.4) | 216 (38) | 25.5 (2.3) | 4.9 (2.4) | 29.3 (12.7) | 262 (8) | 0.18 (0.06) | 9.5 (0.5) | 1.0 (1.0) | 449 (282) | 45 (24) |
|  |  | 20–40 | 6.2 (0.2) | 415 (41) | 28.7 (1.2) | 12.9 (6.2) | 13.0 (6.6) | 277 (29) | 0.35 (0.17) | 11.2 (1.7) | 2.2 (1.8) | 685 (286) | 48 (51) |
|  |  | 40–60 | 6.5 (0.4) | 303 (162) | 19.4 (7.7) | 18.8 (13.4) | 4.7 (2.5) | 315 (44) | 0.75 (0.83) | 9.3 (5.1) | 1.7 (1.2) | 744 (182) | 855 (586) |
|  | S12 | 0–20 | 5.9 (0.3) | 197 (18) | 26.0 (2.1) | 6.2 (4.1) | 194.9 (117.4) | 277 (29) | 0.29 (0.54) | 9.9 (0.7) | 1.1 (1.3) | 745 (374) | 190 (54) |
|  |  | 20–40 | 6.5 (0.8) | 296 (79) | 29.0 (8.9) | 12.0 (9.1) | 89.2 (166.9) | 283 (25) | 0.30 (0.51) | 11.3 (2.1) | 1.8 (1.5) | 592 (370) | 76 (38) |
|  |  | 40–60 | 6.5 (0.3) | 201 (70) | 15.1 (6.6) | 11.0 (11.3) | 5.6 (4.5) | 350 (89) | 0.21 (0.32) | 8.9 (4.8) | 1.9 (2.0) | 463 (205) | 792 (374) |
|  | S14 | 0–20 | 6.0 (0.3) | 451 (36) | 22.0 (3.1) | 1.1 (0.0) | 198.0 (49.6) | 320 (60) | 0.22 (0.06) | 9.5 (0.5) | 1.8 (2.1) | 989 (735) | 270 (50) |
|  |  | 20–40 | 6.3 (0.3) | 597 (36) | 20.8 (5.0) | 2.9 (2.3) | 37.3 (25.0) | 273 (27) | 0.32 (0.06) | 11.0 (1.3) | 1.3 (1.1) | 649 (366) | 98 (47) |
|  |  | 40–60 | 6.5 (0.5) | 455 (141) | 13.6 (7.3) | 3.5 (3.2) | 6.0 (3.1) | 353 (58) | 0.24 (0.14) | 7.9 (2.6) | 1.0 (1.2) | 566 (307) | 1,158 (644) |
| PC | J12 | 0–20 | 5.9 (0.1) | 264 (58) | 27.7 (3.0) | 11.5 (4.4) | 20.7 (14.6) | 285 (54) | 1.03 (0.50) | 8.8 (0.8) | 0.4 (0.4) | 275 (120) | 125 (61) |
|  |  | 20–40 | 6.0 (0.3) | 448 (54) | 28.7 (1.6) | 13.2 (4.4) | 11.0 (8.1) | 247 (35) | 0.99 (0.40) | 11.6 (2.8) | 1.4 (1.5) | 762 (373) | 93 (43) |
|  |  | 40–60 | 5.9 (0.7) | 250 (98) | 17.0 (7.6) | 6.8 (3.5) | 5.0 (4.9) | 275 (68) | 2.01 (1.98) | 8.4 (6.3) | 4.8 (5.8) | 1,898 (1,716) | 842 (371) |
|  | S12 | 0–20 | 5.8 (0.1) | 240 (32) | 27.5 (1.6) | 11.5 (3.2) | 9.6 (7.4) | 285 (45) | 0.14 (0.08) | 9.1 (0.8) | 0.7 (0.5) | 312 (119) | 145 (35) |
|  |  | 20–40 | 5.9 (0.3) | 381 (27) | 27.5 (0.8) | 18.0 (5.0) | 3.5 (3.7) | 270 (20) | 0.21 (0.18) | 11.7 (2.2) | 1.6 (1.3) | 768 (322) | 154 (89) |
|  |  | 40–60 | 6.4 (0.7) | 220 (74) | 14.5 (6.7) | 12.0 (6.0) | 3.9 (6.1) | 295 (96) | 0.10 (0.06) | 8.2 (6.5) | 2.1 (1.9) | 973 (463) | 993 (420) |
|  | S14 | 0–20 | 5.8 (0.2) | 483 (48) | 23.5 (2.8) | 1.2 (0.4) | 51.2 (30.6) | 293 (38) | 0.14 (0.02) | 9.4 (1.2) | 1.8 (1.1) | 808 (349) | 156 (72) |
|  |  | 20–40 | 5.9 (0.4) | 609 (114) | 22.0 (1.5) | 3.1 (3.1) | 23.7 (7.7) | 285 (15) | 0.19 (0.06) | 11.6 (2.7) | 2.2 (1.7) | 1,080 (501) | 200 (193) |
|  |  | 40–60 | 6.0 (0.7) | 313 (78) | 11.7 (4.0) | 3.8 (3.5) | 4.4 (3.9) | 313 (127) | 0.13 (0.03) | 8.0 (5.3) | 2.8 (3.1) | 1,284 (861) | 1,202 (380) |
| PF | J12^a^ | 0–20 | 6.0 (0.4) | 284 (67) | 26.2 (3.5) | 8.5 (5.9) | 16.2 (11.8) | 268 (69) | 1.31 (0.68) | 8.9 (1.2) | 0.6 (0.6) | 364 (183) | 238 (288) |
|  |  | 20–40 | 6.2 (0.4) | 444 (44) | 28.8 (1.6) | 16.7 (10.9) | 20.1 (15.6) | 253 (39) | 1.53 (0.56) | 10.9 (2.5) | 1.2 (1.4) | 753 (467) | 70 (16) |
|  |  | 40–60 | 6.0 (0.5) | 310 (113) | 23.0 (6.5) | 10.5 (10.3) | 12.9 (9.6) | 292 (35) | 1.41 (0.51) | 11.2 (6.9) | 4.2 (3.7) | 1,597 (1,021) | 588 (350) |
|  | S12 | 0–20 | 6.0 (0.3) | 275 (60) | 26.8 (2.6) | 12.1 (1.6) | 12.0 (12.7) | 283 (57) | 0.11 (0.04) | 9.1 (1.0) | 0.8 (1.0) | 426 (229) | 202 (78) |
|  |  | 20–40 | 6.1 (0.3) | 360 (89) | 28.3 (1.2) | 19.0 (5.1) | 1.9 (1.5) | 280 (14) | 0.10 (0.04) | 11.7 (2.6) | 1.7 (1.6) | 740 (436) | 155 (100) |
|  |  | 40–60 | 6.2 (0.4) | 223 (90) | 15.7 (7.5) | 15.1 (9.8) | 1.9 (2.4) | 295 (41) | 0.14 (0.14) | 9.4 (7.2) | 2.0 (1.6) | 844 (496) | 723 (354) |
|  | S14 | 0–20 | 6.0 (0.3) | 445 (173) | 17.8 (3.7) | 1.1 (0.0) | 88.8 (38.3) | 310 (26) | 0.15 (0.08) | 9.2 (1.4) | 1.0 (0.5) | 567 (204) | 392 (206) |
|  |  | 20–40 | 6.2 (0.4) | 510 (95) | 20.8 (1.6) | 2.5 (1.6) | 29.0 (9.1) | 277 (30) | 0.13 (0.05) | 11.4 (2.2) | 1.5 (1.0) | 747 (304) | 183 (152) |
|  |  | 40–60 | 6.2 (0.5) | 274 (96) | 9.1 (4.7) | 7.3 (8.9) | 3.8 (2.0) | 345 (75) | 0.11 (0.02) | 8.6 (5.9) | 2.1 (2.2) | 854 (754) | 1,153 (401) |

UC, uncultivated control soils; UF, uncultivated fertilised soils; PC, *Phalaris* cultivated control soils; PF, *Phalaris* cultivated fertilised soils; J12, June 2012; S12, September 2012; S14, September 2014; DOC, dissolved organic carbon; TN, total nitrogen; TP, total phosphorous; TS, total sulphur; TK, total potassium.

^a^ Before the first fertilisation occasion.
