# Supplementary material for: Impact of Reed Canary Grass Cultivation and Mineral Fertilisation on the Microbial Abundance and Genetic Potential for Methane Production in Residual Peat of an Abandoned Peat Extraction Area
Source: PLoS One. 2016 Sep 29;11(9):e0163864. doi: 10.1371/journal.pone.0163864 (PMC5042519; doi:10.1371/journal.pone.0163864)
Supplement: S3 Table — (DOCX) [file pone.0163864.s005.docx]

**S3 Table. Statistically significant relationships (linear mixed-effects modeling) between different gene parameters and soil chemical variables, and between gene parameters of different treatments in three layers.**

| **Layer (cm)** | **Gene parameters** | **Soil chemical parameters** | | | | | | | | | | |  | | **Cultivation** | |  | | **Fertilisation** | | |
| --- | --- | --- | --- | --- | --- | --- | --- | --- | --- | --- | --- | --- | --- | --- | --- | --- | --- | --- | --- | --- | --- |
|  |  | pHH_2_O | DOC | TN | NH_4_-N | NO_3_-N | TP | PO_4_-P | TS | SO_4_-S | Ca | TK | |  | | P (vs. U) | |  | | UF (vs. UC) | PF (vs. PC) |
| 0–20 | Bact | −2.08* |  |  |  |  |  |  |  |  |  |  | |  | |  | |  | |  |  |
|  | Arch |  |  |  |  |  |  |  | 2.89** | 2.18* | −2.90** |  | |  | |  | |  | |  |  |
|  | Arch% |  | −2.24* |  | 2.49* |  |  |  | 4.18*** | 2.40* | −2.95** | −2.14* | |  | |  | |  | |  |  |
|  | mcrA |  |  | 2.25* | 2.29* |  |  |  |  |  |  |  | |  | |  | |  | |  |  |
|  | mcrA% |  |  |  |  |  |  |  | −2.59* |  |  |  | |  | |  | |  | |  |  |
| 20–40 | Bact |  | 2.24* |  |  |  |  |  |  |  |  |  | |  | |  | |  | |  |  |
|  | Arch |  |  |  |  |  | 2.20* |  |  |  |  |  | |  | |  | |  | |  |  |
|  | Arch% |  |  |  |  |  | 4.48*** |  | 3.30** |  |  |  | |  | |  | |  | |  |  |
|  | mcrA | 3.46** | 2.87** |  |  | −2.83* |  |  |  |  |  |  | |  | |  | |  | |  |  |
|  | mcrA% |  |  |  |  |  |  |  |  |  |  |  | |  | |  | |  | |  |  |
| 40–60 | Bact | −2.31* |  |  |  |  | 2.34* |  |  |  |  |  | |  | |  | |  | |  |  |
|  | Arch |  |  |  |  |  | 3.11** | −3.28** |  |  |  |  | |  | |  | |  | |  | 2.06* |
|  | Arch% |  |  | −2.47* |  |  |  | −3.14** | 3.54** |  |  |  | |  | |  | |  | |  | 2.49* |
|  | mcrA |  | 2.64* |  |  |  | 4.29*** |  |  |  |  |  | |  | |  | |  | |  | 2.53* |
|  | mcrA% |  |  |  |  |  |  | 2.92** |  |  |  |  | |  | |  | |  | |  |  |

P, *Phalaris* cultivated soils; U, uncultivated soils; UC, uncultivated control soils; UF, uncultivated fertilised soils; PC, *Phalaris* cultivated control soils; PF, *Phalaris* cultivated fertilised soils; Bact, bacterial 16S rRNA gene abundance; Arch, archaeal 16S rRNA gene abundance; Arch%, archaeal proportion in prokaryotes community; mcrA, *mcrA* abundance; mcrA%, *mcrA* proportion in archaeal community; DOC, dissolved organic carbon; TN, total nitrogen; TP, total phosphorous; TS, total sulphur; TK, total potassium.

* – p ˂ 0.05; ** – p ˂ 0.01; *** – p ˂ 0.001
