# Supplementary material for: Impact of Reed Canary Grass Cultivation and Mineral Fertilisation on the Microbial Abundance and Genetic Potential for Methane Production in Residual Peat of an Abandoned Peat Extraction Area
Source: PLoS One. 2016 Sep 29;11(9):e0163864. doi: 10.1371/journal.pone.0163864 (PMC5042519; doi:10.1371/journal.pone.0163864)
Supplement: S4 Table — (DOCX) [file pone.0163864.s006.docx]

**S4** **Table. Statistically significant relationships (Spearman’s Rank correlations) between different gene parameters (n=12; measurements of September 2012 and 2014), and between gene parameters (measurements of September 2012 and 2014) and means of the study years vegetation periods` soil physical parameters as well as methane emissions (n=6 in all cases) in three layers of the studied soil groups.**

| **Soil group** | **Layer (cm)** | **Gene parameters** |  | **Gene parameters** | | |  | **Physical parameters** | |  | **CH_4_ emission** |
| --- | --- | --- | --- | --- | --- | --- | --- | --- | --- | --- | --- |
|  |  |  |  | Arch | mcrA | mcrA% |  | SoilT | WT |  |  |
| UC | 0–20 | Bact |  |  |  |  |  | 0.83* |  |  |  |
|  |  | mcrA% |  | – | – | – |  | 0.83* |  |  |  |
|  | 20–40 | Arch |  | – | 0.64* | – |  |  | 0.94** |  |  |
|  |  | Arch% |  | – |  | – |  |  | 0.83* |  |  |
|  |  | mcrA |  | 0.64* | – | – |  |  |  |  | 0.94** |
|  | 40–60 | Bact |  |  | 0.72** |  |  |  |  |  |  |
|  |  | mcrA |  | 0.59* | – | – |  | 0.83* |  |  | 0.83* |
|  |  | mcrA% |  | – | – | – |  | 0.89* |  |  |  |
| UF | 0–20 | Bact |  |  | 0.71** | 0.73** |  |  |  |  |  |
|  |  | Arch% |  | – | −0.65* | – |  | −0.94** |  |  |  |
|  | 20–40 | Arch% |  | – | 0.59* | – |  |  |  |  |  |
|  |  | mcrA |  | 0.59* | – | – |  |  |  |  | 0.94** |
|  | 40–60 | Bact |  | 0.68* | 0.77** |  |  |  |  |  |  |
|  |  | mcrA |  | 0.69* | – | – |  |  |  |  | 0.94** |
|  |  | mcrA% |  | – | – | – |  | 0.83* |  |  |  |
| PC | 0–20 | Bact |  |  | 0.76** |  |  | 0.90* |  |  | −0.89* |
|  |  | Arch% |  | – | −0.66* | – |  |  | 0.94** |  |  |
|  |  | mcrA |  |  | – | – |  | 0.81* | −0.83* |  | −0.94** |
|  |  | mcrA% |  | – | – | – |  |  | −0.94** |  |  |
|  | 20–40 | Bact |  | 0.58* |  |  |  |  |  |  |  |
|  |  | Arch% |  | – |  | – |  |  | −0.89* |  |  |
|  |  | mcrA |  |  | – | – |  | 0.94** |  |  |  |
|  |  | mcrA% |  | – | – | – |  |  | −0.89* |  |  |
|  | 40–60 | Bact |  | 0.76** | 0.94*** |  |  |  |  |  |  |
|  |  | Arch% |  | – |  | – |  | −0.83* |  |  |  |
|  |  | mcrA |  | 0.82** | – | – |  |  |  |  |  |
| PF | 0–20 | Bact |  |  |  |  |  |  |  |  | −0.89* |
|  |  | Arch% |  | – | −0.73** | – |  |  | 0.94** |  |  |
|  |  | mcrA |  |  | – | – |  |  | −0.83* |  | −0.83* |
|  |  | mcrA% |  | – | – | – |  |  | −0.94** |  |  |
|  | 20–40 | Bact |  | 0.59* |  |  |  |  |  |  |  |
|  | 40–60 | Arch% |  | – |  | – |  |  |  |  | 0.94** |
|  |  | mcrA |  | 0.76** | – | – |  |  |  |  |  |
|  |  | mcrA% |  | – | – | – |  | 0.83* | −0.94** |  |  |

UC, uncultivated control soils; UF, uncultivated fertilised soils; PC, *Phalaris* cultivated control soils; PF, *Phalaris* cultivated fertilised soils; Bact, bacterial 16S rRNA gene abundance; Arch, archaeal 16S rRNA gene abundance; Arch%, archaeal proportion in prokaryotes community; mcrA, *mcrA* abundance; mcrA%, *mcrA* proportion in archaeal community; SoilT, soil temperature; WT, groundwater depth. Dash (–) denotes “not tested”.

* – p ˂ 0.05; ** – p ˂ 0.01; *** – p ˂ 0.001
